# Cell–Cell Contact Mediates Gene Expression and Fate Choice of Human Neural Stem/Progenitor Cells

**DOI:** 10.3390/cells11111741

**Published:** 2022-05-25

**Authors:** William B. McIntyre, Mehran Karimzadeh, Yasser Riazalhosseini, Mohamad Khazaei, Michael G. Fehlings

**Affiliations:** 1 Institute of Medical Sciences, University of Toronto, Toronto, ON M5S 1A8, Canada; brett.mcintyre@mail.utoronto.ca; 2 Krembil Research Institute, University Health Network, Toronto, ON M5T 0S8, Canada; 3 Department of Human Genetics, McGill University, Montreal, QC H3B 1S6, Canada; mehran.karimzadeh@uhnresearch.ca (M.K.); yasser.riazalhosseini@mcgill.ca (Y.R.); 4 McGill University Genome Centre, Montreal, QC H3A 0G1, Canada; 5 Division of Neurosurgery, Department of Surgery, University of Toronto, Toronto, ON M5T 2S8, Canada

**Keywords:** cell density, hippo-signaling, neuronal differentiation, neural stem/progenitor cells, NOTCH, pro-neuronal transcription factors, RNA-sequencing, WNT

## Abstract

Transplantation of Neural Stem/Progenitor Cells (NPCs) is a promising regenerative strategy to promote neural repair following injury and degeneration because of the ability of these cells to proliferate, migrate, and integrate with the host tissue. Precise in vitro control of NPC proliferation without compromising multipotency and differentiation ability is critical in stem cell maintenance. This idea was highlighted in recent clinical trials, where discrepancies in NPC culturing protocols produced inconsistent therapeutic benefits. Of note, cell density plays an important role in regulating the survival, proliferation, differentiation, and fate choice of stem cells. To determine the extent of variability produced by inconsistent culturing densities, the present study cultured human-induced pluripotent NPCs (hiPSC-NPCs) at either a low or high plating density. hiPSC-NPCs were then isolated for transcriptomic analysis or differentiation in vitro. Following sequencing analysis, genes involved in cell–cell contact-mediated pathways, including Hippo-signaling, NOTCH, and WNT were differentially expressed. Modulation of these pathways was highly associated with the regulation of pro-neuronal transcription factors, which were also upregulated in response to higher-density hiPSC-NPC culture. Moreover, higher plating density translated into a greater neuronal and less astrocytic differentiation in vitro. This study highlights the importance of precisely controlling culture conditions during the development of NPC transplantation therapies.

## 1. Introduction

Understanding mechanisms involved in central nervous system (CNS) development can facilitate the development of translationally relevant regenerative cell strategies. Self-renewal, proliferation, and neurogenesis of progenitor cells relies on extracellular paracrine signalling and cell–cell contact. The cell–cell interaction mediated by integral membrane proteins is critical in proliferation, differentiation, and maintenance of stem cells. This is partly due to lateral inhibition mechanisms, involving the release of delta-1 (DLL1) from progenitors, which activate NOTCH-signaling pathways in neighbouring cells [1]. In contrast, the inhibition of NOTCH signaling can halt proliferation and promote neurogenesis. Conceptually, these developmental cues could influence NPCs in vitro to alter their fate choice when applied as a transplantation therapy for the regenerating CNS. A robust example of this is the inhibition of NOTCH signaling in cultured progenitor cells using the γ-secretase inhibitor, DAPT. Functionally, in vitro DAPT treatment can promote a neurogenic fate in both mouse and human embryonic [2,3,4] and induced pluripotent stem cells (iPSCs) [5].

Physical contact-mediated developmental cues can potentially be applied in the same manner to bias progenitor cells towards a particular cell fate. For example, plating embryonic stem cells (ESCs) at a higher localized cell density is necessary to define neuroectodermal specification [6], possibly regulated by the expression of cell-adhesion molecules, like connexin-43 [7]. This is evident in other forms of culture, such as 3D or sphere-forming cultures, where ESCs left to grow longer form denser neurospheres and acquire a greater competency to differentiate into neurons [8]. Exemplifying this, directional growth of neural stem/progenitors (NPCs) towards maturing neurons can be altered through micro-alterations to in vitro plating conditions [9], which may occur through cellular protrusions that deliver NOTCH and/or Ephrin ligands.

The potential benefit of transplanting NPCs into zones of neural injury is based on the cell’s ability to self-renew and proliferate along a neural fate *in vivo*. This is important for several reasons, as the neural injury milieu contains an abundance of cellular debris, reactive neural cells, and reactive oxygen species (ROS), which do not favour transplanted cell survival [10,11]. This is confounded by transplanting post-mitotic neuronal populations that either do not survive [10], or require further optimization to preserve survivability prior to transplantation [12]. Moreover, transplantation of NPCs can promote plasticity by sustaining a developmental transcriptome in spared, injured tissue [13]. In the context of spinal cord injury, harsh injury microenvironments can sometimes promote a more neuroglial fate, as it is difficult to regulate the differentiation capacity of NPCs post-transplantation. Differentiated astrocytes may in some cases be conducive to facilitating regeneration of neural tissue through the formation of the glial scar [10]. However, the scar also serves as a barrier to plasticity, repair, and regeneration. Thus, a more equal proportion of neurons, astrocytes, and oligodendrocytes is required to promote a permissive regenerative microenvironment, promote synaptic plasticity and integration, and replenish the lost cellular niche.

Thus, the culturing microenvironment and cell–cell contact can strongly regulate a variety of processes, including NPC gene expression, fate determination, and commitment to a post-mitotic fate. In the present study, we have analyzed the effect of high density versus low density culture conditions on the transcriptomic changes of human induced pluripotent stem cells (hiPSC)-NPCs. A detailed examination of transcriptomic changes after high vs. low density hiPSC-NPCs prior to differentiation suggests NOTCH, WNT, pro-neuronal, and growth factor signaling may be responsible for biasing NPCs towards a neurogenic cell fate. These findings address the drastic changes observed under variable culture conditions, thereby highlighting the need for standardized manufacturing protocols to produce more consistent cell-based therapeutics.

## 2. Materials and Methods

### 2.1. Experimental Design

Human induced pluripotent stem cells (hiPSC) were thawed and used for this study. Using neural-induction methods described below, cells were plated at either a low or high density for 7 days. Following this, high/low-density hiPSC-NPCs were either differentiated in vitro (*n* = 3 each) to assess their differentiation capacity or extracted from the plate for RNA isolation and subsequent whole-exon sequencing. Significant biological pathways and cellular components were determined using the Panther Enrichment online tool for further discussion.

### 2.2. hiPSC-NPC Culture

The hiPSC line BC1 [14] was obtained from the NIH Center for Regenerative Medicine. hiPSCs were maintained on a feeder-free layer condition with Matrigel (Corning; AZ, USA) in mTeSR1 media (STEMCELL Technologies; Canada), where only passage numbers 30–36 were used for subsequent culture and experiments. hiPSCs were differentiated towards a neural progenitor lineage (hiPSC-NPC) using dual SMAD inhibition in a monolayer. Prior to differentiation (day 0), hiPSCs were dissociated into a single-cell suspension and plated as a monolayer at a density of 20,000 cells/cm^2^ in mTeSR1 media. Cells were left to proliferate to 90% confluency, where media was gradually changed over 2 days using a 1:1 ratio of neural induction medium (NIM) and DMEM/F12 media (supplemented: B27, N2, FGF at 10 ng/mL; 10 μM TGFβ inhibitor at SB431542; 200 ng/mL Noggin; and 3 μM GSK3β inhibitor (CHIR99021). After 7 days in culture, neural rosettes formed and were manually collected and plated in a single cell colony on poly-L-Lysine (PLL)/Laminin-coated dishes in NPC expansion media (NEM; supplemented with B27, N2, FGF at 10 ng/mL and EGF at 20 ng/mL for two passages). Following expansion, cells were then cultured at 1 cell/uL (1000 cells/mL) to form clonal primary neurospheres. To expand each clonal spere, cells were plated in PLL/Laminin-coated 24-well plates (Millipore Sigma; Canada). Three separate lines (denoted as 15, 16, and 150 in raw data files) of hiPSC-NPCs was derived with this method.

### 2.3. Plating Density and In Vitro Differentiation

To study the effect of culture density on the fate determination of NPC lines in vitro, hiPSC-NPCs were cultured for 7 days on Matrigel-coated dishes at the seeding density of 0.05 × 10^6^/mL (for low density culture condition) and 0.25 × 10^6^/mL (for high density culture condition) in NEM. To determine differentiation capacity, cells from both conditions were detached using TrypLE (ThermoFisher Scientific, Waltham, MA, USA) and plated at the same seeding density of 0.05 × 10^6^/mL in NEM without growth factors (no FGF2/EGF) and treated with 10 µg/mL heat-inactivated FBS for 28 days. Half of the media was changed with fresh media every 3 days.

### 2.4. Immunocytochemistry

Following in vitro differentiation, cells on coverslips were fixed for 20 min with a 1:1 ratio of 500 uL 8% paraformaldehyde (PFA) in PBS to 500 uL of cultured media (4% PFA final). Following fixation, cells were permeabilized in 0.1% Triton-X-100 in PBS for 3 min on ice, then placed in blocking solution (5% bovine serum albumin; BSA). Primary antibodies (Table 1) were diluted in blocking solution and incubated overnight at 4 °C. Following incubation, cells were washed 3 × 10 min in 1× PBS and incubated with secondary antibodies at a 1:1000 dilution (Alexa fluor-488, 568, or 647). Cell profile (%) was assessed by counting total cells (DAPI+) by the positive stain for each cell type.

### 2.5. RNA Isolation and Sequencing

rRNA-depleted stranded libraries for each condition (three biological replicates for each high-density cultures and low-density cultures) were generated and multiplexed. Paired-end 100 bp sequencing was performed using the Illumina HiSeq 2000 platform at McGill Genome Centre. In all, 60–70 million sequencing reads were obtained for each sample. Raw sequence reads were aligned to the UCSC human reference genome (hg19) using TopHat with default parameters. To count the mapped reads, HTSeq was used with the reference genome annotation (USCS, hg19).

### 2.6. Sequencing Analysis

The Illumina TruSeq adapters from FASTQ files were trimmed with Trim Galore (version 0.4.4, Felix Krueger, Cambridge, UK) [15]. The STAR software (version 2.6.0c, Alexander Dobin, Cold Spring Harbor, NY, USA), specifying options--outFilterMultimapNmax 2--genomeSAindexNbases 6--alignSJoverhangMin 8--alignSJDBoverhangMin 4--outFilterMismatchNoverReadLmax 0.05 was used to align the FASTQ files to the GRCh38 reference genome [16]. We used StringTie [17] (version 1.3.3b, Mihaela Pertea, Baltimore, MD, USA) quantified TPM for genes in the GRCh38 annotation. EdgeR [18] was used for identifying differentially expressed genes using the raw count of genes.

#### 2.6.1. GO Enrichment Analysis

Gene IDs were uploaded to the Gene set enrichment analysis online PANTHER enrichment tool [19]. PANTHER Overexpression Test (Released: 7 April 2020). Homo sapiens were used as the reference organism, and functional classification was analyzed as either a gene list or visual bar/pie chart. GO categories included Molecular Function, Cellular Components, Biological Process, Protein Class, and Pathway.

A list of NOTCH signaling pathway signatures according to the Reactome database [20] was compiled through MSigDB v6.1 [21]. For each pathway, the enrichment score of differentially expressed genes among low- and high-density cells within that pathway was calculated using SeqGSEA (v1.26.0) [22] log fold change (log2FC), and false discovery rate (FDR) was calculated relative to gene expression of low density-cultured NPCs. Thus, changes in gene expression describe what occurs when NPCs are plated in a higher density culture. Differentially expressed genes were initially sorted if their FDR < 0.05. Subsequently, up and downregulated genes were selected for their log2FC values, where values of 1.5 and above, or −1.5 and below, were selected for enrichment analysis.

#### 2.6.2. KEGG Enrichment Analysis

The KEGG enrichment analysis was applied to find the pathways that were significantly enriched in the differentially expressed transcripts compared with the whole genome. Pathways with a Q ≤ 0.05 were defined as significantly enriched pathways in the differentially expressed transcripts. Significant pathway enrichment was able to identify the most important biochemical metabolic pathways and signal transduction pathways involved in the differentially expressed transcripts.

#### 2.6.3. Assessment of Cell Lineage Gene Programs from RNA-Seq Data

To understand the cellular components of NPCs with different plating density, we used custom gene set enrichment using GSEA. To evaluate gene programs in bulk RNA seq samples, a reference panel of 44 gene signatures was assembled using data from 2 published scRNaseq atlases [23,24] spanning different stages of neural stem/progenitor cell differentiation. In addition, a data set of 67 gene sets was used to deconvolute excitatory and inhibitory neuronal subtypes [25]. Gene set enrichment analysis (GSEA) was performed with these signatures as inputs applied to the bulk RNA-seq for cells cultured in high and low density. Using the GSEA, the normalized enrichment scores (NES) were computed by normalizing enrichment to the average enrichment of 10,000 random gene samples. Adjusted *p* values (FDR) < 0.25 were considered significantly enriched.

### 2.7. RT-qPCR Validation

RNA isolation was completed from identical samples used for sequencing analysis. Briefly, complimentary DNA (cDNA) was synthesized using the Bioline sensiFAST cDNA Synthesis Kit (Cat. No. BIO-65053; FroggaBio, ON, Canada.). All RNA samples were diluted to identical concentrations and subsequently synthesized on a LifePro Thermal Cycler (BIOER) as per the manufacturer’s protocol. TaqMan probes were obtained from ThermoFisher Scientific and selected under Human (Hs) species, ensuring probes spanned all exons. TaqMan Probes *MAP2* (Hs00258900), *ASCL1* (Hs04187546), *NEUROG1* (Hs01029249), *BDNF* (Hs0718934) *GFAP* (Hs00909233), *AQP4* (Hs0024342), *OLIG2* (Hs00300164), *DLK1* (Hs05635283), *DLL4* (Hs00184092), *NOTCH1* (Hs0106014), *NOTCH2* (Hs01050702) *NFIA* (Hs00325656), *NFIB* (Hs01029174), *MSI1* (Hs01045894), and *GAPDH* (Hs02786624) were used for validation. Relative Quantification (RQ) was calculated based on ∆∆Ct, where fold-change of gene expression was subsequently calculated using 2^−∆∆Ct^. The housekeeping gene *GAPDH* was used as the inter-sample control for relative Ct quantity present in each sample. Low-density NPC ∆Ct values were averaged for each gene, where they were compared to each biological replicate in high-/low-density samples (N = 3 per group). Log2 fold-change (Log2FC) was used to scale all samples to a similar-sized y-axis, where it was calculated by taking the log2 of the 2^−∆∆Ct^ value (fold-change).

### 2.8. Statistical Analysis

Differentiation profiles and RT-qPCR validation of high vs. low hiPSC-NPCs were assessed using an unpaired two-way Student’s *t*-test. The significance level of all analyses was set at *p* < 0.05. Statistical analysis was conducted, and graphical presentation was prepared in R (version 3.6.1). Fisher’s exact test was used to determine the presence of any non-random associations in comparison to the homo sapiens (all genes in database) reference.

## 3. Results

### 3.1. Differentiation Capacity of High- vs. Low- Density hiPSC-NPCs

**Figure 1:**
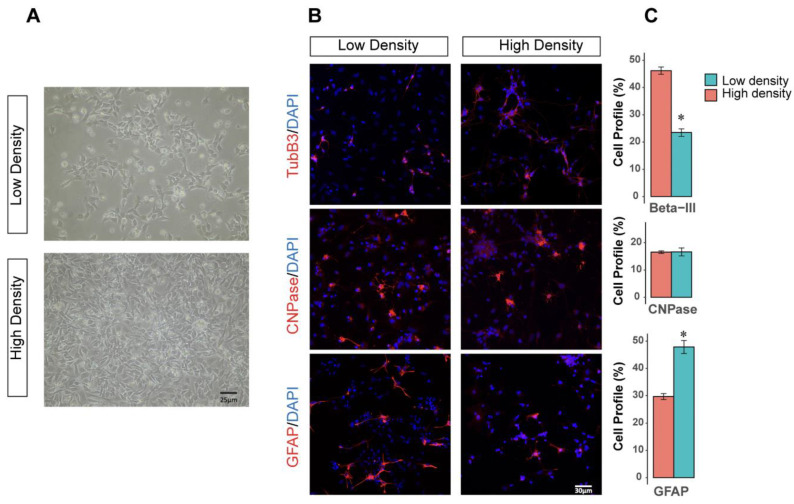
Tripotency of hiPSC-NPCs at low and high densities. (**A**) Visual representation of high vs. low density hiPSC-NPCs prior to cellular differentiation. (**B**) Immunocytochemistry of differentiated hiPSC-NPCs (blue = DAPI; red = cell marker). (**C**) Cell profile (%) of total cells (DAPI+) as a ratio of cell marker + cells. * = *p*-value <0.05, N = 3 per group.

To explore the effect of culture density on the differentiation of NPC lines, we differentiated cells in the presence of FBS. Differentiating NPC lines for four weeks resulted in their differentiation to neurons (βIII-tubulin+), astrocytes (GFAP+), and oligodendrocytes (CNPAse+), confirming their tripotency. In high-density culture, NPCs differentiated to more neurons (46.2.7 ± 2.3%,) compared to low density culture (23.4 ± 2.3%, *p* < 0.5). However, culturing in a low-density condition resulted in the differentiation of NPCs to more astrocytes (47.8.4 ± 4.1%) compared to NPCs cultured in high density (29.6.29 ± 1.9% *p* < 0.05) (Figure 1).

### 3.2. Plating Density Alters Transcriptional Programs in hiPSC-NPCs

**Figure 2:**
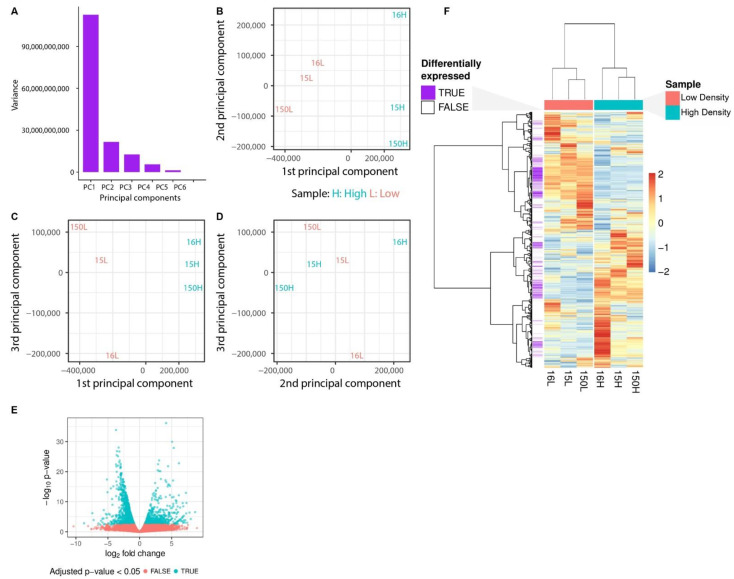
High- and low-density cells exhibit distinct transcriptomic profiles. (**A**) Distribution of variance among the principal components of the fragments per million reads (FPKM) matrix of gene expression. (**B**) Scatterplot of the first two principal components of the FPKM matrix of gene expression. Orange: low density cells. Turquoise: high density cells. (**C**) Scatterplot of the first and third principal components of the TPM matrix of gene expression. (**D**) Scatterplot of the second and third principal components of the TPM matrix of gene expression. (**E**) Volcano plot for differential expression analysis comparing high density cells with low density cells. Orange: genes that adjusted *p*-values did not pass the cut-off of 0.01. Turquoise: genes with adjusted *p*-value < 0.05. (**F**) Heatmap with unsupervised clustering of genes and samples. Values indicate z-score of gene’s FPKM values among the samples. Distance metric: 1-Pearson correlation. Clustering algorithm: ward [26]. Purple: genes with adjusted *p*-value < 0.05. White: genes which do not pass the adjusted *p*-value cut-off of 0.05.

To compare the global transcriptome of NPCs cultured in low vs. high density, we performed RNA-seq analysis (Figure 2). Despite the considerable similarity of gene expression patterns in these two conditions, we identified some important differences. A total of 1239 genes were significantly upregulated (FDR > 0.05, log2FC > 1.5) and 1009 genes were significantly downregulated (FDR > 0.05, log2FC < (−1.5)). There was increased expression of NOTCH, WNT, pro-neuronal/differentiation signaling, and decreased expression of FGF/EGF-related signaling in NPCs grown in high density compared to NPCs grown in low density.

### 3.3. Functional Enrichment Analysis of Differentially Expressed Genes

**Figure 3:**
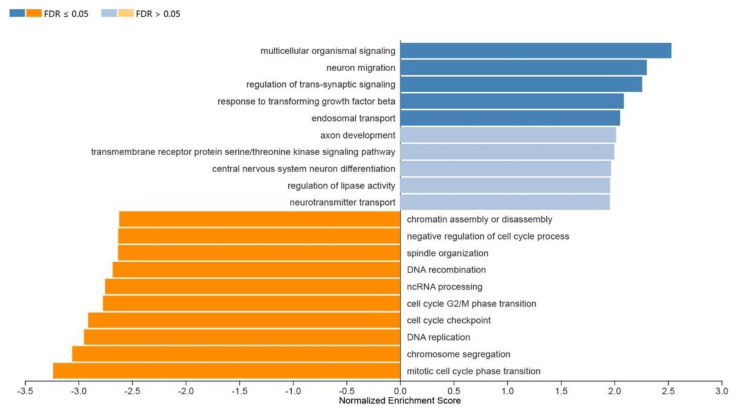
Normalized enrichment scores of 10 of the most upregulated (blue) and downregulated (orange) GO terms.

**Figure 4:**
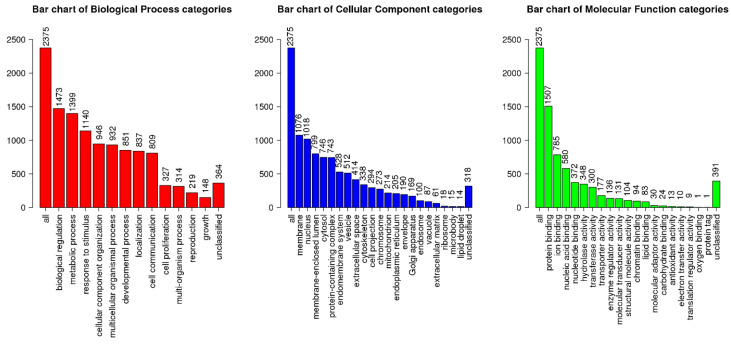
Most affected biological processes, cellular components, and molecular function categories from GO enrichment terms.

**Figure 5:**
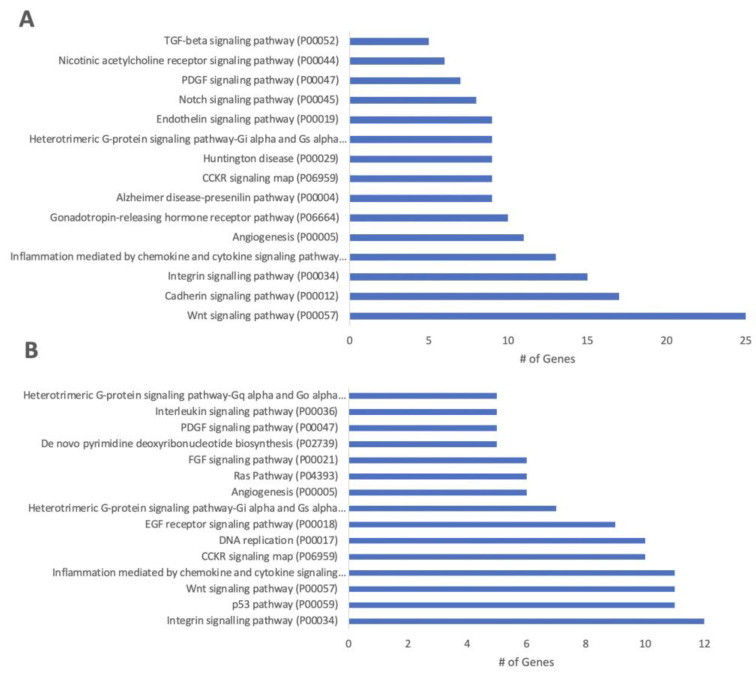
Upregulated (**A**) and downregulated (**B**) enriched pathways of high vs. low density hiPSC-NPC cultures. Expression was determined relative to low-density cultures. The number of genes were determined from PANTHER analysis.

To investigate how the differentially expressed genes (DEGs) in our dataset related to translated biological events, GO enrichment analysis was performed using PANTHER. Thus, the differential expression of clustered genes is ultimately representative of functional pathways. From PANTHER, enriched biological processes, cellular components, and molecular function terms were identified (Figure 3, Figure 4 and Figure 5). DEGs in a higher density related to functional TGFβ-signaling, neuronal migration, and synaptic signaling. The most enriched numbers of DEGs were associated with TGFβ, NOTCH, growth factor (EGF, FGF), Wnt, Cadherin, and integrin signaling pathways.

**Figure 6:**
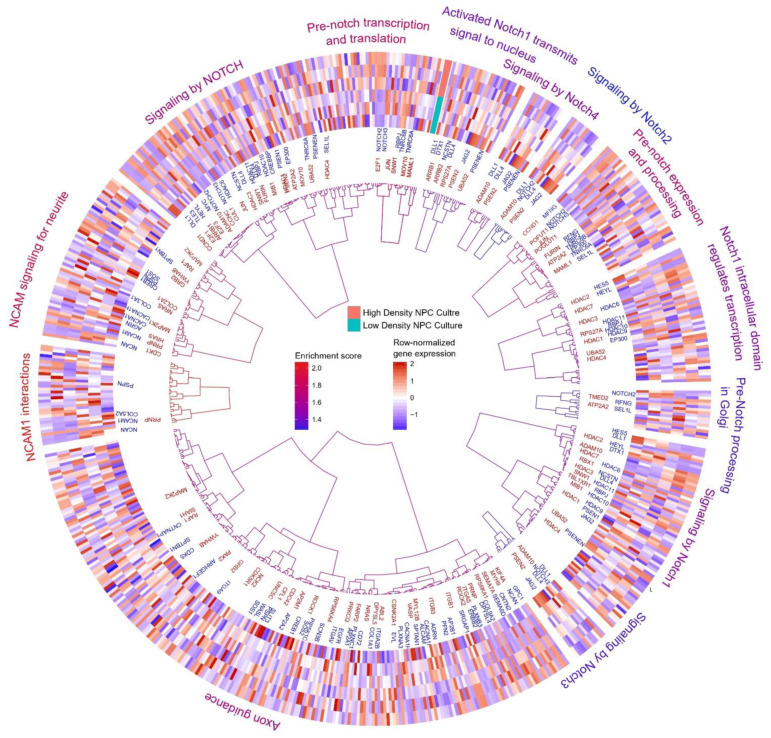
Differential activity of NOTCH signaling among low -and high-density cells. We hierarchically clustered genes within each NOTCH signaling signature MSigDB Reactome (version 6.1, San Diego, USA) according to their transcription in low- and high-density samples. The colour of the dendrograms shows the pathway enrichment score. We plotted the name of each significantly upregulated gene (red) and downregulated gene (blue) next to the dendrogram. The circular heatmap shows the z-score of normalized gene expression.

For each NOTCH signaling gene signature, as annotated through MSigDB Reactome database (version 6.1, San Diego, USA), we clustered differentially expressed genes (Figure 6). We discovered differential activity of the NOTCH signaling cascade among the low- and high-density cells according to the transcriptome data.

### 3.4. Assessment of Culture Density on Biasing Cell Lineage Specification

**Figure 7:**
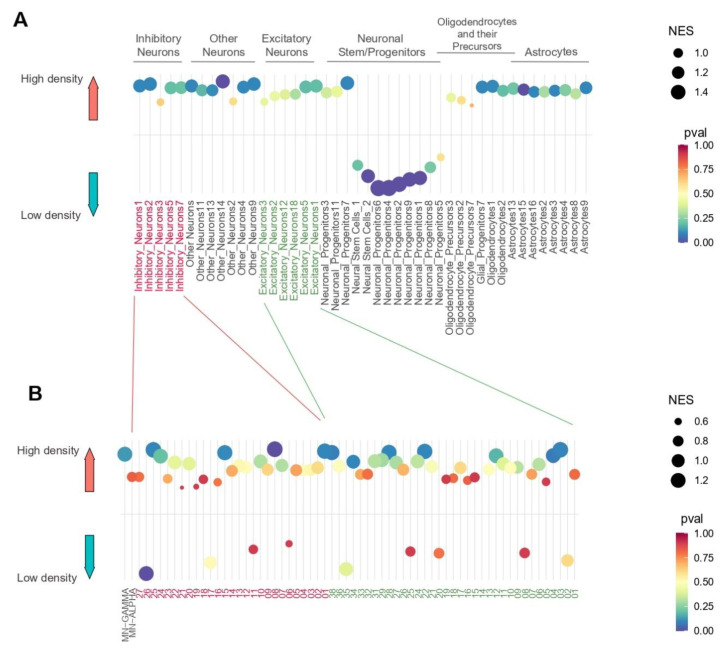
Comparisons of GSEA projections of high- and low-density cells to scRNA-seq atlases of different stages of neural stem/progenitor cell differentiation (**A**) as well as excitatory and inhibitory neuronal subtypes (**B**). Circle diameters indicates the normalized enrichment score (NES); X-axis is the cell types; red and green text highlight inhibitory and excitatory interneurons, respectively.

Next, we used cell type specific gene signatures from published single-cell RNA sequencing atlases [23,24,25] as input for gene-set enrichment analysis (GSEA) (Figure 7A) to characterize cell identity programs enriched/depleted in the NPCs cultured at high- or low-density. Low-density cultured cells demonstrated enrichment for cells that are more at a progenitor state, while cells that were cultured at higher density demonstrated more enrichment for different neural cell types. (Figure 7). Notably, comparisons of our GSEA projections with scRNA-seq databases revealed no significant *p*-values (<0.05) in high-density cultures (Figure 7B). However, the scRNA-seq comparison data used for this analysis are in reference to post-mitotic, mature neural cell types. As such, trends towards significance in these groups are likely indicative of the transcriptomic bias of high-density cultures towards the indicated lineage.

## 4. Discussion

NPCs can alter their fate choice in response to the microenvironmental cues provided during their culture and propagation. However, the extent to which cultured cells process the microenvironmental cues from cell–cell contact to their fate commitment remains poorly understood. To address this, we investigated the effects of prolonged contact-mediated interactions of hiPSC-NPCs by plating cells at either a lower or higher density (five-fold increase in cells/mL). RNA-sequencing of hiPSC-NPCs prior to differentiation suggests plating at a higher cell density can increase the propensity of NPCs to differentiate into neurons through modulation of cell-adhesion mediated Hippo-signaling, which affects downstream NOTCH, WNT, and pro-neuronal signaling.

### 4.1. Growth Factors and Sprouty

**Figure 8:**
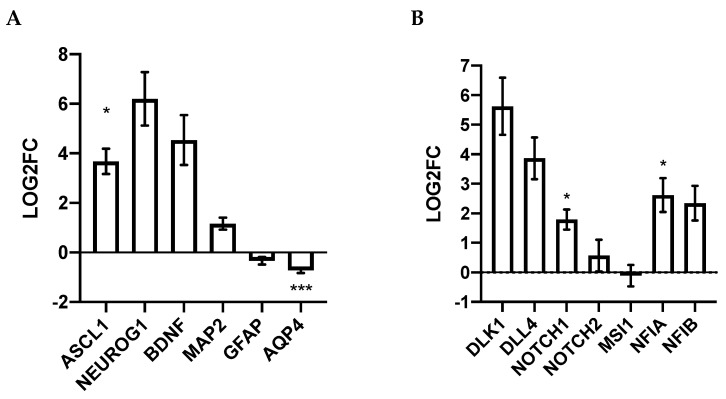
Sequencing validation of neuron, astrocyte, and oligodendrocyte differentiation markers (**A**) as well as NOTCH signaling markers (**B**) expressed in high-/low-density neural stem/progenitor cells. (**A**) RT-qPCRs for transcripts that promote neurogenesis (NEUROG1, ASCL1, BDNF), mature neuronal markers (MAP2), astrocytic markers (GFAP, AQP4, NFIA, NFIB), and oligodendrocytes (OLIG2). (**B**) Gene expression of notch ligands (DLK1, DLL4), followed by NOTCH receptors (NOTCH1, NOTCH2), and negative regulators (MSI1) of NOTCH signaling. LOG2FC was calculated relative to low-density hiPSC-NPCs. Statistical significance was determined using an unpaired Student’s *t*-test (* *p*-value < 0.05, *** *p*-value < 0.001).

The downregulation of several Sprouty genes (SPRY1, 2, and 4) is particularly interesting in the context of neuronal differentiation. Typically, SPRY is a regulator of both FGF/EGF signaling, which is associated with increased progenitor cell growth and reduced neural differentiation [27,28]. Notably, higher density culturing can prolong early phases of the cell cycle (G1), which makes progenitor cells more responsive to differentiation ques [29,30]. Notably, higher density culturing increased BDNF expression (Figure 8). SPRY2, a negative regulator of BDNF, was downregulated in higher density. As a result, downregulation of SPRY2 can promote neuronal differentiation possibly through BDNF-mediated effects [31]. In a similar fashion, suppression of SPRY4 leads to a reduction in cellular proliferation and stemness marker expression [32]. Thus, increased cell density can restrict SPRY2 and SPRY4 expression, which promotes both general and neuronal-specific cellular differentiation, respectively. Lower plating density also suggests the maintenance of a stem/progenitor-like transcriptome (Figure 7A).

### 4.2. Differential Wnt Signaling in High/Low Cell Density

**Figure 9:**
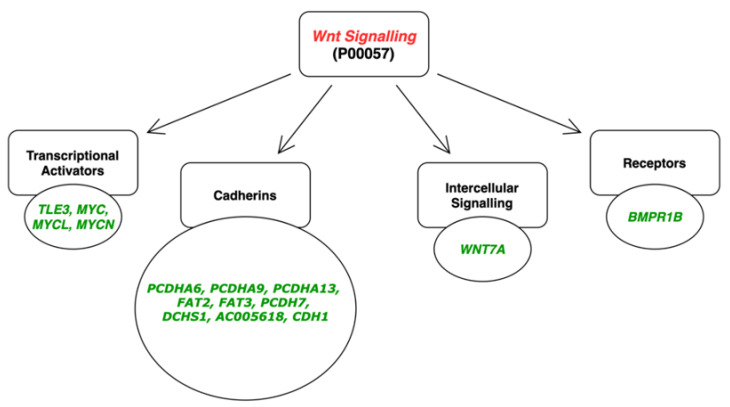
Major components of the Wnt signalling pathway upregulated by high density neural progenitor cell culture. Pathway analysis completed with PANTHER. GO: P00057.

In a culture dish, plating with adherent substrates can alter the differentiation propensity of NPCs through altered expression of laminin, integrin, and cadherin molecules [33]. Wnt and adhesion molecule signaling are closely intertwined, as they have the same intracellular regulator, β-catenin (Figure 9). Within this study, the Wnt ligand, WNT7A was upregulated, whereas WNT7B was downregulated following high-density culturing. Recombinant WNT7A administered in a culture system can increase the number of immature neurons, which mediates neurogenesis through a canonical, β-catenin-dependent Wnt pathway [34,35]. Interestingly, over-expressing WNT7B in the developing forebrain can impair neuronal differentiation by modulating downstream pro-neuronal transcription factors [36]. These effects may be time-point dependent, as WNT7A can variably induce or prevent neurogenesis [37]. Downstream of Wnt signaling, targeted transcription factors, LHX2 and CER1, are enhanced in response to higher progenitor density. Interestingly, LHX2 is known to regulate critical steps in neural development by activating the Wnt antagonist CER1. CER1 is an antagonist to Wnt, which promotes neuronal differentiation by inhibiting non-neuronal (mesodermal/endodermal) lineages [38]. Altogether, modulated activity of WNT7 molecules and their downstream effectors may be utilized to promote a neurogenic fate in cultured hiPSC-NPCs.

### 4.3. Higher Culture Density Promotes Hippo-Related Signaling

To acquire a terminal fate, neural cell differentiation will coincide with the arrest of cellular proliferation. Contact inhibition (CI) and subsequent hippo-signaling is a process by which cellular proliferation is halted once physical contact is mediated by the clustering of neighbouring cells. At high cell densities, a key component of the hippo pathway, YAP/TAZ, is phosphorylated and translocated out of the nucleus, thus relaying neuroepithelial differentiation and impedance of pluripotency. Dense cell clustering antagonizes intracellular hippo signaling, which is a constitutively active self-renewal pathway mediated by transmembrane junctions [39], integrins [40], and cadherins [41]. Notably, our results coincide with this process, as higher density downregulated several downstream hippo-target genes: PTPN14, TEAD4, CRIM1, and NT5E [42]. Reduced expression of PTPN14 and TEAD4 is indicative of the downregulation of hippo-signaling; PTPN14 promotes YAP localization in the nucleus via phosphatase activity [43], whereas TEADs are the downstream transcription factors that bind with YAP/TAZ to promote cellular proliferation [44,45].

Higher density also upregulated several mediators of hippo-signaling inhibition: Fibronectin 1 (FN1), E-Cadherin (CHD1), atypical FAT cadherins (FAT2/3), and MST1; the former three gene groups being important for inhibiting hippo-signaling via cell-mediated contact, and the latter being an essential component of the hippo-kinase core responsible for the inactivation of canonical hippo signaling [39,46]. Importantly, the relationship between CI and neuronal differentiation has been described previously. The modulation of hippo-signaling in primary murine and immortalized SH-SY5Y cell cultures suggest that hippo promotes sonic hedgehog and antagonizes NOTCH/Wnt/B-catenin signaling pathways [47,48,49]. Typically, activation of the hippo-kinase core, and subsequent inhibition of downstream YAP/YAZ alleviates the inhibition of several proneuronal genes (ASCL1, NEUROG1/2, NEUROD1) [50]. This suggests that during high cell plating density, CI-mediated hippo inhibition can promote various forms of neurogenesis, thus increasing the propensity of NPCs to differentiate into neurons.

### 4.4. NOTCH Signaling in Higher Density

**Figure 10:**
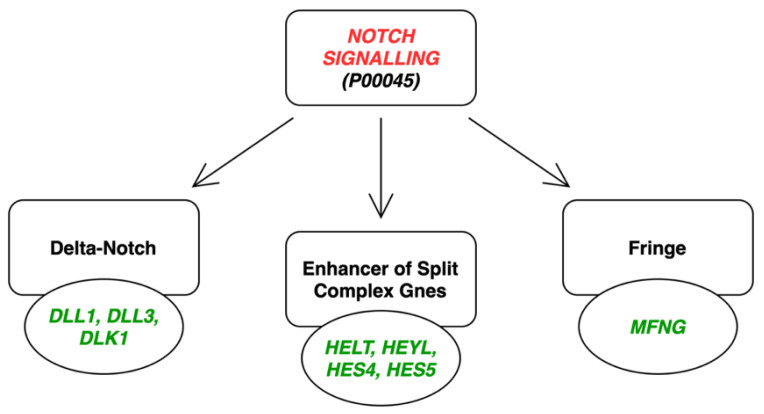
Upregulated effectors of NOTCH and downstream fringe signaling. Pathway analysis completed with PANTHER. GO: P00045.

NOTCH inhibition in vitro is a common technique that can commit human embryonic stem cells towards neurogenesis [2]. However, higher density cultures may promote a similar dynamic process in which the NOTCH signaling mediates the transition between proliferation and neurogenesis through intermediate or transitional progenitor stages. Notably, Delta (DLL)-1/3 and Delta-like (DLK)-1 NOTCH ligands exhibited increased expression following culturing in high density (Figure 10). This is seemingly contradictive, as DLL1 agonizes and DLK1 antagonizes the NOTCH-1 receptor. However, increased DLL1 expression in progenitors may represent “transitional zone state”. During in vivo neurogenesis, DLL1 is expressed in an oscillatory manner, where sustained expression promotes expression of proneuronal genes (ASCL1/NEUROG1) to affirm neural cell fate [51]. Through well-established lateral inhibition-mediated mechanisms, DLL1 can promote sustained expression of itself through an intrinsic neurogenic pathway via negative feedback, while inhibiting differentiation in neighbouring cells. As a result, a ratio of cells in the neurogenesis transitional zone will yield 1 neuron: 1 maintained progenitor [1]. Thus, upregulation of DLL1, as well as its downstream effector HES5, in this study suggests high density progenitors exhibit similarities to neurogenic progenitors in the transitional zone of development.

Notably, DLK1 was the most upregulated and significantly enhanced gene (11.4 log2FC: FDR < 10–12) following high culturing density in the progenitor stage. In a culturing dish, DLK1 administration can enhance neuron production from human neural stem cells by antagonizing the NOTCH ligands Delta and Jagged [52]. Downstream of NOTCH signaling and the regulation of HES transcription factors can modulate progenitor cell differentiation. In the context of a transplantation therapy in neural injury, DLK1 knockout in transplanted progenitor cells reduces an NPC’s ability to differentiate into neurons through a growth-factor-mediated pathway [53]. Notably, HES6 was upregulated after high density culturing, which can promote neuronal differentiation by forming an inhibitory heterodimer with HES1, a key promoter of proliferation and inhibitor of neurogenesis [54]. Further evidence of NOTCH inhibition is exhibited through downregulated NRARP and HES7, which are typically activated downstream of NOTCH signaling [55].

Altogether, the simultaneous upregulation of DLK1 expression is important for the arrest of proliferation, while sustained DLL1/3 expression is important for the transition from self-renewal to neurogenesis. This suggests that higher density cultures may recapitulate a transitional zone of neurogenesis, which provides crucial information regarding the maintenance of unlimited self-renewal and proliferation of hiPSC-NPCs in culture.

### 4.5. Higher Plating Density Promotes Neuronal and Prevents Astroglial Fate

**Figure 11:**
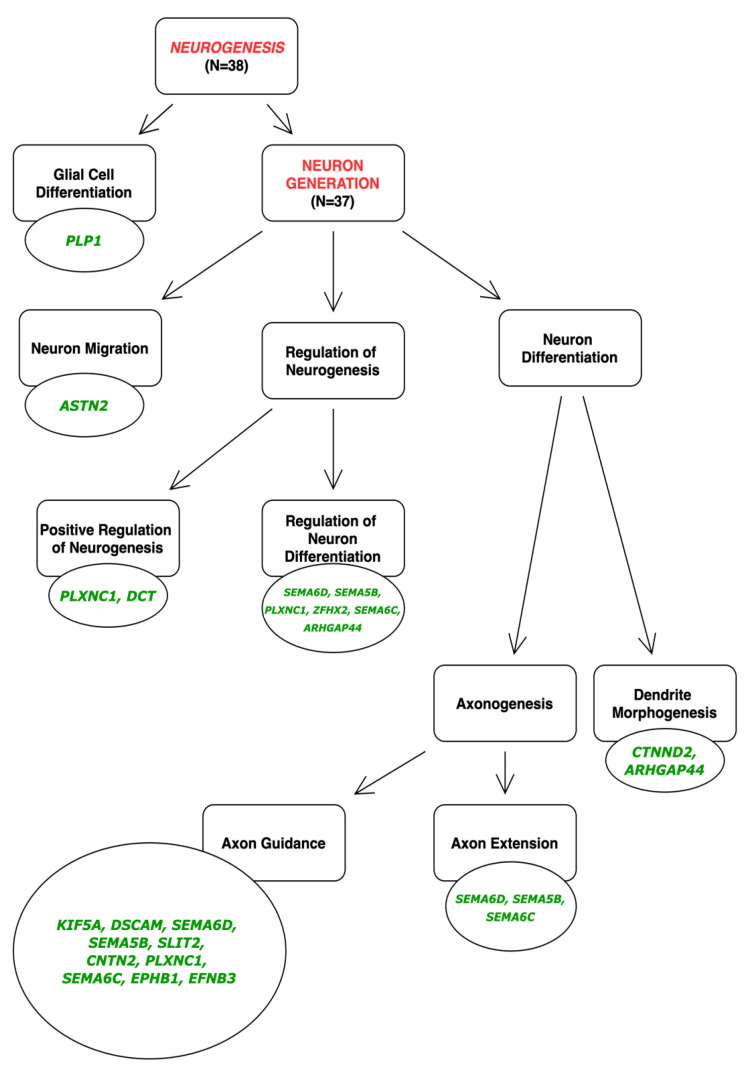
Upregulated pathways associated with neurogenesis in a high-density neural progenitor cell culture. Molecules and transcription factors involved in neuronal differentiation, production of axons (axonogenesis; morphogenesis), and subsequent guidance ques are all significantly upregulated. Pathway analysis completed with PANTHER.

As the inhibition of NOTCH and subsequent downstream pathways suggest proliferation is inhibited, it is likely that the gene expression of differentiation markers is upregulated. As such, markers of the three major cell types of the CNS (astrocytes, oligodendrocytes, and neurons) support their tripotent differentiation potential (Figure 1). High-density plated progenitors exhibited increased expression of Aquaporin-4 (AQP4) and GFAP, which linked to increase parallel expression levels following the onset of stem cell differentiation (Figure 11) [56]. As an indicator of oligodendrocyte maturation, high-density cells exhibited increased expression of proteolipid protein 1 (PLP1) and Selenoprotein P (SEPP1) (Figure 11), which are both required for oligodendrocytes and myelinate cells. Higher plating density also reduces the expression of both OLIG1/2, which are involved in early- and late-state oligodendrocyte differentiation [57]. OLIG1/2 downregulation may be the result of NOTCH signaling inhibition [58], which can significantly reduce OLIG1/2-related signaling in the developing brain. Most notably, however, these changes in gene expression do not reflect the functional consequences of oligodendrocyte formation, which is unaffected by high-density culturing (Figure 1C).

Increased levels of expression of several pro-neuronal genes corroborates enhanced neurogenic differentiation of high-density progenitor cultures. Increased ASCL1 expression following high-density culturing in this study is a robust indicator of enhanced neurogenesis. ASCL1 is a pioneer transcription factor that is necessary to promote NPC proliferation, as well as the specification of neurogenic cell fates through increased chromatin accessibility and de novo gene expression [59,60]. NEUROG1/2 expression was also increased in high density cultures, which are proneuronal transcription factors that can initiate neuronal differentiation [61,62,63]. Not only can it promote the differentiation capacity into neurons, but NEUROG1 signaling simultaneously inhibits gliogenesis by sequestering the gliogenic-Smad1/CBP/p300 activating complex away from STAT-promoter sites, and towards neurogenic transcriptional activation. Notably, in higher levels of NEUROG1 expression, bone morphogenic protein (BMP) signaling can promote neurogenesis, whereas lower NEUROG1 expression can promote gliogenesis. This coincides with our data, in which BMP Receptor Type IB (BMPR1B) was upregulated. Notably, this receptor is sensitive and specific to BMP ligands [64]. Overall, enhancement of neurogenic pathways and inhibition of gliogenic pathways are present within our dataset.

#### Cell Density Biases Interneuron Fate

Bulk RNA-sequencing was conducted on cell groups, which cannot be distinguished by cell type without single-cell isolation. However, bulk-RNA-seq data can be deconvoluted to identify the distinct transcriptomic information of cell types [65]. Interestingly, slight variances were observed in signatures that cluster interneuron subtypes (Figure 7B). Interneuron development from hiPSC-derived cells is a relatively new idea in the literature, but nonetheless presents a potentially beneficial role of interneurons in recovery spasticity and neuropathic pain observed after spinal trauma [66]. Exemplifying the importance of cell density, an exact seeding density of 25,000 cells/cm^2^ can result in a greater proportion of CHX10+ expressing V2a interneurons [5]. A similar protocol developed from the same group also suggests CHX10 expression may be dependent on the initial seeding density [67].

### 4.6. RT-qPCR Validation Corroborates Functional Immunostaining

Notably within our study, several RT-qPCR results did not corroborate RNA-sequencing results. Firstly, *NOTCH1* expression was not significantly altered in RNA-seq but was upregulated in higher-density culture when analyzed using RT-qPCR (Figure 8). The use of a housekeeping gene to normalize expression, as well as significant intra-sample variability between cell lines used as biological replicates for this study, may explain other inconsistencies between RT-qPCR and sequencing results (Table S1. Extensive intra-cell line variability in transcript abundance may also explain why several NOTCH and pro-neuronal factors (DLK1, NEUROG1, ASCL1, etc.) were trending, but not statistically significant (Figure 8A,B). Most notably, the two astrocytic markers *GFAP* and *AQP4* were upregulated in higher density cultures according to sequencing results, but either trended or were significantly downregulated in higher density through RT-qPCR analysis (Figure 8A). Corroborating the idea that sequencing may not always align with RT-qPCR results, Everaert et al., compared the typical RT-qPCR benchwork to five sequencing workflows. Surprisingly, 15–20% of protein-coding genes represented discordant or non-matching results observed between sequencing and RT-qPCR platforms [68]. In general, RT-qPCR and sequencing have comparable accuracies, but benchwork RT-qPCR is much more specific as it is slightly biased towards commercially available probes. Probes used in this study for *AQP4* and *GFAP* span exon junctions and thus will not detect contaminant genomic DNA. The specificity of the RT-qPCR assay may detect more translationally active exon variants and thus may reflect more functionally relevant molecules. Corroborating this, the RT-qPCR data matches the functional immunocytochemistry assessment that suggests high density promotes a more neuronal (TUBB3+) and less astrocytic (GFAP+) differentiation profile (Figure 1).

### 4.7. Application to Transplantation Studies

Neuronal maturation in a dish only partially reflects the developmental ques that determine cell fate *in vivo*. When developing iPSC cultures in bulk, protocols must be standardized to ensure consistent cellular differentiation within and between manufacturing laboratories. This idea is exemplified through transcriptomic changes in differentiated neural progeny from human hiPSCs, which suggests “tuning” WNT signaling at an early stage of differentiation can help reduce variability [69]. This is especially important, as large-scale manufacturing of hiPSC lines can produce variability in cultured cells, potentially altering their efficacy in recent clinical trials [70,71]. Culturing recommendations to produce and expand hiPSC-NPCs do not account for cell density. At the time of transplantation, cells are typically thawed and immediately transplanted. In the context of spinal cord injury, a higher cell dosage (>500 k cells total) at the point of engraftment can promote a greater extent of neuronal differentiation when directly compared with lower doses (<100 k cells total) [72,73,74]. Altogether, maintaining a higher density of cells can promote a more pronounced neurogenic fate of hiPSC-NPCs used for transplantation therapy.

## 5. Conclusions

Apart from high-density culturing prior to the mass expansion and freezing of cell lines, combinatory strategies involving chemical and physical modulation of NOTCH signaling may be able to promote neurogenic cell fate to a greater extent. These strategies can combine biochemical alterations with increased cell density to promote a greater neurogenic fate. Beneficial evidence of the physical and chemical modulation of cell-fate is observed in well-established culturing protocols, such as retinoic-acid induced-neural progenitor differentiation [75] and dual-SMAD inhibition to promote neurogenic specification of iPSCs [12]. Ultimately, the pathways outlined in our analysis highlight several mechanisms that are crucial for in vitro hiPSC-NPC differentiation. Most importantly, large discrepancies in fate choice, as well as transcriptomic differences suggest that standardizing culturing density can help produce more consistent hiPSC-NPC therapeutics.

## Figures and Tables

**Table 1:** Antibodies used to assess differentiation profile of cultured hiPSC-NPCs.

PROBE (Stock; Cat No.)	Dilution	Target
β-III-Tubulin (BioLegend; MMS-435P)	1:500	Immature neurons
GFAP (Abcam; ab33922)	1:500	Astrocytes
CNPase (Abcam; ab6319)	1:500	Oligodendrocytes

## Supplementary Materials

The following supporting information can be downloaded at: https://www.mdpi.com/article/10.3390/cells11111741/s1, File S1. Raw count matrix and differential gene expression (DGE) analysis files. Each sample was labelled with either “L” or “H,” indicating high- or low-density plated cells, respectively. Numbers on experimental groups indicate a different source (plate) the plated cells were derived from. Notably, each sample was derived from the same passage and cell line. Unanalyzed data can be derived from the raw count matrix tab. Table S1. Plating density alters gene expression in a pairwise manner
