# Supplementary material for: Cell–Cell Contact Mediates Gene Expression and Fate Choice of Human Neural Stem/Progenitor Cells
Source: Cells. 2022 May 25;11(11):1741. doi: 10.3390/cells11111741 (PMC9179342; doi:10.3390/cells11111741)
Supplement: Supplementary file 1 [file cells-11-01741-s001.zip › Supplemental Files/Supplementary Table S1.pdf]

Supplementary Table S1. Plating density alters gene expression in a pairwise manner. Samples X15L/H and X150L/H represent hiPSC-NPCs plated from different passages, whereas X16L/H represent a second hiPSC-NPC line curated from a different round of dual-SMAD inhibition. Fold change (LogFC) may not accurately represent proportional gene expression changes, as averages between all “L” and “H” samples were taken. As a result, false-discovery rate (FDR) and logFC are representative as groups and not intra-sample changes in gene expression. For example, X15L and X16H have similar values between high/low density groups, but their proportional change within identical samples plated at different densities (X16L & X16H) exhibit a more substantial change.

**Gene of Interest**	**X15L**	**X150L**	**X16L**	**X15H**	**X150H**	**X16H**	**LogFC**	**FDR**
AQP4	26	31	716	522	1430	12464	4.48	.01
GFAP	226,946	43,028	272,415	589,617	701,928	427,442	1.83	.007
NOTCH1	8787	5805	5570	11,468	11,640	9061	0.84	.065
NEUROG1	0	0	2	18	12	48	5.26	<0.001
DLK1	0	0	0	183	237	934	11.98	<0.001
